# Rescue of mutant gonadotropin-releasing hormone receptor function independent of cognate receptor activity

**DOI:** 10.1038/s41598-020-67473-w

**Published:** 2020-06-29

**Authors:** Emery Smith, Jo Ann Janovick, Thomas D. Bannister, Justin Shumate, Vadivel Ganapathy, Louis Scampavia, Timothy P. Spicer

**Affiliations:** 1 Department of Molecular Medicine, The Scripps Research Molecular Screening Center, Scripps Research Florida, 130 Scripps Way #1A1, Jupiter, FL 33458 USA; 2 Department of Chemistry, The Scripps Research Molecular Screening Center, Scripps Research Florida, 130 Scripps Way, Jupiter, FL USA; 3 Texas Tech University Health Sciences Center, 3601 4th Street, Lubbock, TX USA

**Keywords:** Cell biology, Cell signalling, Protein folding, Protein transport

## Abstract

Molecules that correct the folding of protein mutants, restoring their functional trafficking, are called pharmacoperones. Most are clinically irrelevant and possess intrinsic antagonist or agonist activity. Here, we identify compounds capable of rescuing the activity of mutant gonadotropin-releasing hormone receptor or GnRHR which, is sequestered within the cell and if dysfunctional leads to Hypogonadotropic Hypogonadism. To do this we screened the E90K GnRHR mutant vs. a library of 645,000 compounds using a cell-based calcium detection system. Ultimately, we identified 399 compounds with EC_50_ ≤ 5 µM with no effect in counterscreen assays. Medicinal chemistry efforts confirmed activity of 70 pure samples and mode of action studies, including radioligand binding, inositol phosphate, and toxicity assays, proved that we have a series of tractable compounds that can be categorized into structural clusters. These early lead molecules rescue mutant GnRHR function and are neither agonist nor antagonists of the GnRHR cognate receptor, a feature required for potential clinical utility.

## Introduction

The gonadotropin-releasing hormone receptor (GnRHR) belongs to a super family of G-protein coupled receptors (GPCRs). There are many mutations across the GnRHR that cause this protein to misfold, not traffic to the plasma membrane, and remain in the endoplasmic reticulum (ER). The quality control system (QCS) of the cell is responsible for the proper production, folding and transport of proteins from the ER to the cell membrane. Endogenous “chaperones” are present to protect the proteins from misfolding but, they are not protein-specific. Many misfolded protein mutants are able to retain (or regain) substantial biological activity but are viewed as inactive in the cell only due to their incorrect cellular location, not because of loss of function^1^. Often times, these misfolded proteins are unable to traffic to the cell membrane. When this occurs, ligands cannot bind to nor activate these proteins, and a physiologic defect occurs^1,2^.

G-protein-coupled receptors are maintained under the QCS machinery. Normally they are produced in the ER and shuttled to the plasma membrane where they become functional with the appropriate ligands. After ligand binding, the WT GnRHR activates the Gα_q_/11 G protein, which activates the inositol phosphate pathway, leading to the release of intracellular calcium which affords us the ability to easily interrogate this target for drug screening^3^. While GnRHR signals primarily through Gα_q_/11 to activate phospholipase C β (PLCβ) it has also been described as coupling through Gα_s_ which drives adenylate cyclase and ultimately cAMP formation thus stimulating PKA activation of CREB. Although gonadotropin promoter subunits contain cAMP response elements, it appears that the MAPK cascade is favored over cAMP pathway for driving gonadotropin promoter activation. This is an important distinction because the MAPK cascade is directly associated to the Gα_q_/11 signaling pathway which is the foundation basis of our assay signaling and detection format^4^.

GnRH is responsible for neural regulation of reproductive function. GnRH enters the portal circulation and binds to a specific receptor (GnRHR) on pituitary gonadotropes, stimulating the release of luteinizing hormone and follicle stimulating hormone. There are 31 different mutations of the GnRHR protein that have been found to lead to misfolding of the protein in which the GnRHR cannot get to the cell surface and bind with GnRH. These mutations cause a disease known as congenital Hypogonadotropic Hypogonadism or Kallmann’s syndrome^3,5,6^.

In this study we utilized the GnRHR protein with the mutation E90K, which is dysfunctional and insensitive to GnRH stimulation when expressed in cells^2,3^. Previously we had developed an assay to discover pharmacoperones capable of rescuing the E90K GnRHR protein, which recover the GnRH stimulation and release of intracellular calcium. We further refined the assay and completed a large-scale high throughput screening (HTS) campaign, testing more than 645,000 compounds. Hits were further validated for GnRHR selectivity by confirmation in an IP-One assay as well as screening against the “+” DOX (addition of doxycycline) counterscreen previously described. Several interesting structural series arose which were subjected to medicinal chemistry analysis. Original hits and structural analogs were obtained and retested. Activity was confirmed in the original assays, and ligand-binding assays proved that we have a series of active and tractable compounds that can be categorized into drug-like structural clusters, compounds which not only rescue GnRHR function but also are neither agonists nor antagonists. Results of this HTS campaign along with the derivation of several potent and selective structural classes that were found in the present study will be discussed here.

## Materials and methods

### Materials

Two compounds that rescue GnRHR function, SR-01000435409 and SR-01000544741 (Deltagen Research Laboratories, San Mateo, CA), were used as positive controls validating the assay for HTS. Note that STK062325 is identical to SR-01000435409 but was purchased from a commercial vendor (Vitas-M, Champaign, IL). Stable cell lines were created in HeLa cells as previously described^2,7^. Fetal bovine serum (FBS) was obtained from HyClone (Logan, UT). The sources of other reagents are as follows: GnRH agonist, d-tert-butyl-Ser6-des-Gly10-Pro9-ethylamide-GnRH (Buserelin; Hoechst-Roussel Pharmaceuticals, Somerville, NJ), myo-[2-^3^H(N)]-inositol (NET-114A; PerkinElmer, Billerica, MA), Iodine-125 (carrier free, NEZ033L; PerkinElmer, Billerica, MA), Dulbecco’s modified Eagle’s medium (DMEM), PBS (GIBCO, Invitrogen).

### HTS optimized primary GnRHR Fluo2 FLIPR pharmacoperone assay

**Figure 1:**
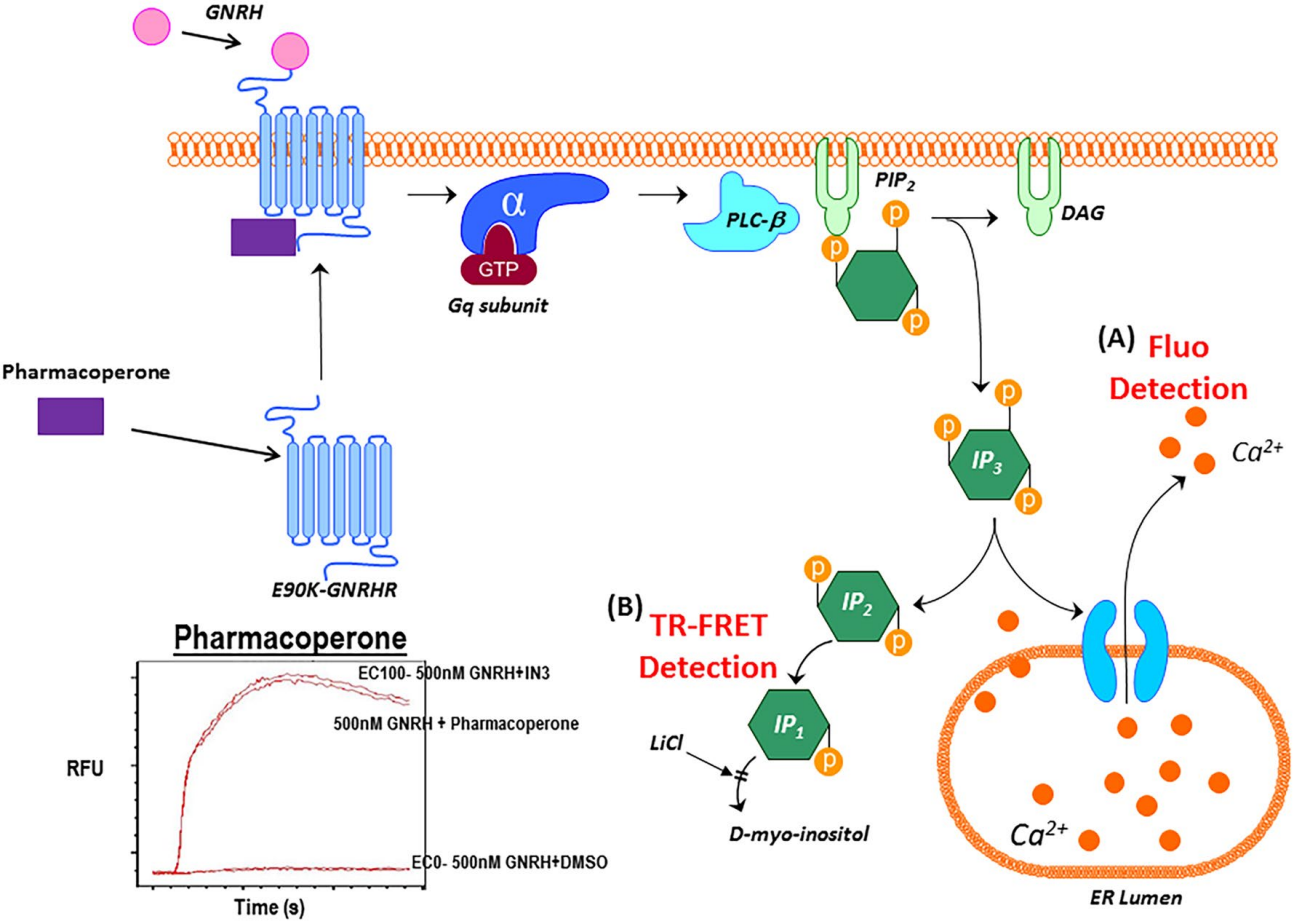
GnRHR pharmacoperone assay principle. (**A**) The GnRHR agonist, GnRH, stimulates the GPCR, activating the Gq subunit to stimulate IP3 to cause a release of intracellular calcium. The fluorescence of the Ca^2+^-sensitive dye Fluo2 is measured in the FLIPR kinetically, with pharmacoperones such as SR-01000435409 causing an increase in RFU signal over time compared to no pharmacoperone. (**B**) The IP-One TR-FRET assay utilizes the same pathway but, detects the binding of IP1 to the tagged antibody. The increase in IP1 present from the activation of Gq by GnRH, with the activity of the pharmacoperone, based on competition for the TR-FRET Ab, results in a decrease the overall TR-FRET signal.

These methods have been adapted from our previous publication and now demonstrate 1536 well miniaturization methods. HeLa cells stably expressing GnRHR E90K mutant under the control of a tetracycline-controlled transactivator (tTA) were cultured in growth media (1 × DMEM + 10% FBS and 1 mg/L gentamicin). On the day of screening, cells were thawed and added to plates (3 μL/well, 1,500 cells/well) in growth media. Note, our former published method used cells that were kept in culture while methods reported here were modified to use freezer ready stocks that were thawed and used as described. Prior to implementing this approach we did compare both methods and outcomes using the two Deltagen control molecules described which reproduced overlapping EC_50_ data for each compound in each condition. Cell addition was followed immediately by pin-tool addition (GNF, San Diego, CA) of test compounds and controls (positive control was 10 μM SR-01000435409; negative control was DMSO only) in 26 nL DMSO. Pintools are stainless steel micro slotted pins in an array that allow for precise and accurate compound or vehicle transfer in the low nanoliter volumetric range from source plates to assay plates. The treated plates were incubated for 17 h at 37 °C and 5% CO_2_ prior to addition of 2 μL of Fluo-2 AM dye. Following a 1 h-incubation at 37 °C , 5% CO_2_ and a 10 min room temperature equilibration with dye, 50 nL of GnRH (500 nM final) was added in a solution of 75%:25% DMSO:H_2_O followed by determination of calcium flux using the FLIPR Tetra. A detailed outline of the primary assay can be found in Supplemental Table 1. An illustration of the primary assay principle is found in Fig. 1.

### HTS optimized secondary GnRHR IP-One TR-FRET pharmacoperone assay

**Figure 2:**
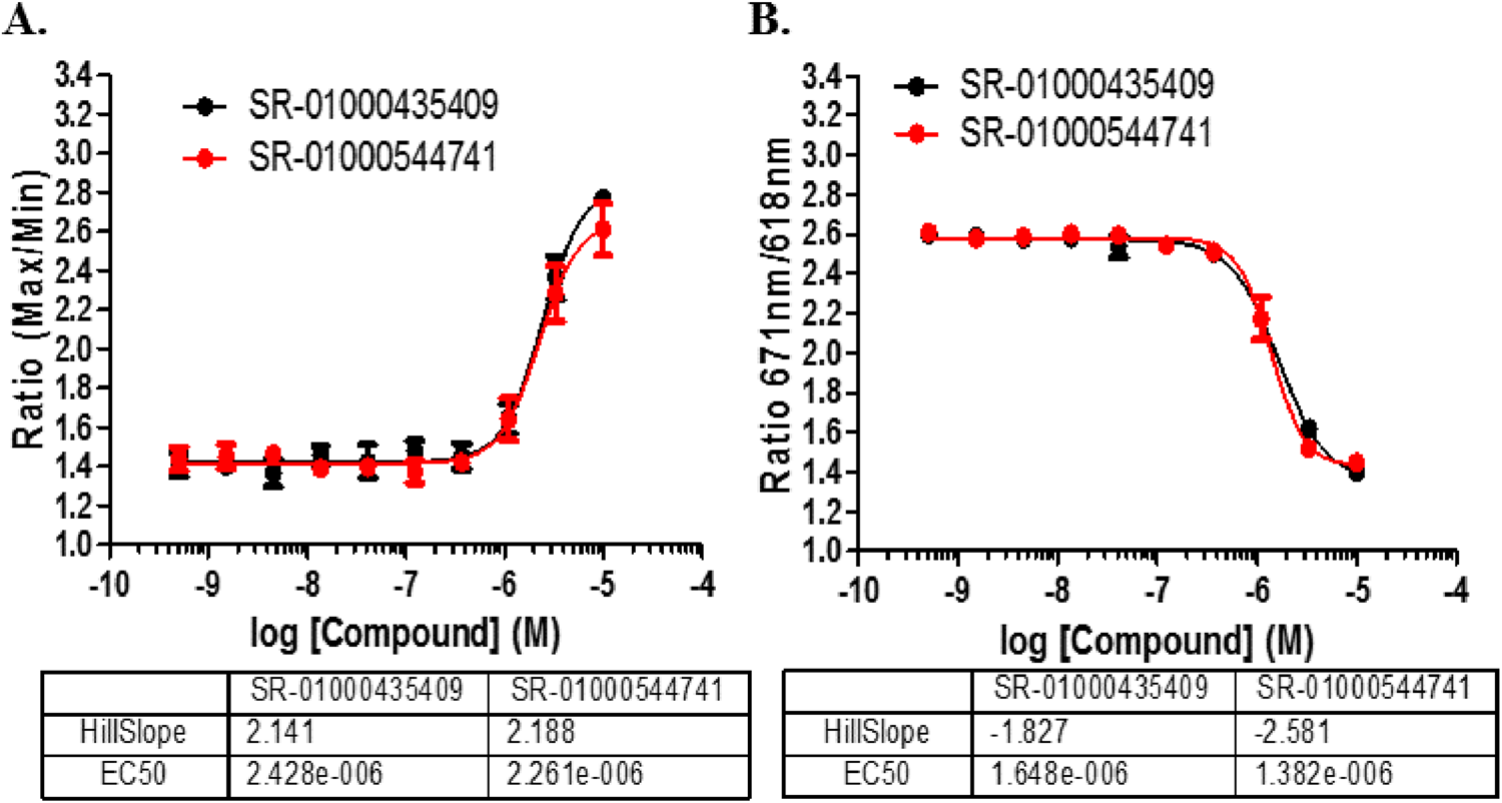
1536-well assay response to GnRHR controls. (**A**) Concentration–response of the controls when tested in the Fluo2 assay vs. (**B**) the IP-One TR-FRET assay. EC_50_ values are shown in M. N = 16 replicates per concentration; error bars presented in SD.

The same cells and controls were utilized as in the primary FLIPR assay. On the day of screening, cells were thawed and added to plates (3 μL/well, 1,500 cells/well) in growth media. This was followed immediately by pin-tool addition of test compounds and controls (positive control was 10 μM SR-01000435409; negative control was DMSO only) in 26 nL DMSO. The plates were incubated for 17 h at 37 °C, 5% CO_2_ prior to addition of 3 μL of 1,000 nM GnRH in 2X stimulation buffer or stimulation buffer only (buffer provided in CisBio IP-One Kit). The plates were incubated for 2 h at 37 °C and 5% CO_2_. 1.5 μL IP-One D2 (1:20 dilution—CisBio) and 1.5 μl IP-One Cryptate (1:20 dilution—CisBio) were added to each well and incubated for 1 h at room temperature in the dark. The plates were read on Viewlux (PerkinElmer Life Sciences) with excitation at 340 nM and emissions at 618 nM and 671 nM. A detailed outline of the confirmation assay can be found in Supplemental Table 2. The concentration responses of the control compounds are shown in Fig. 2.

### *HTS optimized counterscreen of “*+*” DOX GnRHR Fluo2 FLIPR pharmacoperone assay*

The optimized counterscreen was identical to the primary screen described except that cells were cultured in the presence of 1 μg/mL doxycycline for 36 h prior to plating and during all phases of the experiment.

### IP agonist and antagonist determination on wildtype GnRHR HeLa cells: secondary assays

Stably transfected HeLa cells containing the human wildtype (WT) GnRHR were plated (20,000 cells/ well) in 0.25 mL DMEM/10% FBS/20 µg/mL Gentamicin (DFG). The cells were allowed to attach and grow for 51 h in a 48-well plate at 37 °C and 5% CO_2_. Cells were washed twice with 0.5 mL DMEM/0.1% BSA/20 µg/mL Gentamicin (DBG), then preloaded with 0.25 mL of 4 μCi/mL myo-[2-^3^H(N)]-inositol in DMEM (inositol-free medium; IFM) and allowed to incubate for 18 h at 37 °C and 5% CO_2_. Cells were washed two times with 0.3 mL of IFM/5 mM LiCl and treated for 2 h with 0.25 mL of each compound. For agonist activity, 1 µM and 10 µM were tested using the 11 compounds identified from the HTS screen with the STK062325 compound as a control and 1% DMSO containing 100 nM Buserelin as agonist control (Fig. 6A). The compounds were prepared in a final 1% DMSO in IFM/5 mM LiCl.

For antagonist activity, 1 µM and 10 µM were tested using the 11 compounds and STK062325 as a control in the agonist check but were prepared in a solution of IFM/5 mM LiCl containing 1 nM Buserelin (EC_50_ for the wildtype stable cells) for true competition. After 2 h, the media was removed, 0.5 mL of 0.1 M formic acid was added to each well and the cells were frozen. Total IP was determined (6, 7). Data show means (n ≥ 3); SEM are shown (Fig. 6B).

### IP assay to determine rescue on mutant E90K GnRHR HeLa cells

The stably transfected HeLa cells containing the GnRHR mutant E90K were plated in a 48-well plate at 2 × 10^4^ cells per well in 0.25 mL DFG and allowed to attach and grow for 51 h. The cells were washed 2 times with 0.5 ml DBG and a threefold, 10-point concentration–response curve was used for each compound to show rescue and determine EC_50_. All solutions were prepared in 0.25 mL of 4 μCi/ml myo-[2-^3^H(N)]-inositol in DMEM (inositol-free medium; IFM) with a final 1% DMSO and allowed to incubate for 18 h (preload and rescue) at 37 °C. After the 18 h incubation, the cells were washed with DBG two times for 10 min at 37 °C and 20 min at 37 °C to wash out the compounds. The cells were stimulated with 100 nM Buserelin for 2 h at 37 °C. After 2 h, the media was removed, 0.5 mL of 0.1 M Formic Acid was added to each well and the cells were frozen. Total IP was determined (Table 2 and Supplemental Fig. 3). STK062325 was used as positive control. Data show means (n ≥ 3); SEM are shown.

### Binding assays: Scatchard analysis

Stably transfected HeLa cells containing E90K GnRHR mutant were cultured and plated in growth medium as described above, except 5 × 10^4^ cells in 0.5 mL of growth medium were added to 24-well Costar cell culture plates. Fifty-one hours after plating, cells were washed twice with 0.5 mL of DBG, and then 0.5 ml of DBG containing 5 µM of each compound and STK062325 as positive control in 1% DMSO (final) was incubated for 18 h at 37 °C. Cells were washed 2 × 10 min at 37 °C, then 1 × 20 min at 37 °C with DBG containing 1% DMSO. Then the cells were quickly washed twice with DMEM/ 0.1%BSA/10 mM HEPES. A range of concentrations of ^125^I-Buserelin (2.5 × 10^5^ to 8 × 10^6^ CPM/mL), prepared in our laboratory, was added in 0.5 mL of the same medium to the cells. Cells were incubated at room temperature for 90 min^8^. After 90 min, the media was removed, cells washed 2× with 1 ml ice cold PBS and radioactivity was measured as previously described^9^. To determine nonspecific binding, the same concentrations of radioligand were added to the cells in the presence of 10 µM unlabeled GnRH. Saturation binding curve fits and calculations were computed using SigmaPlot 12.5 (Jandel Scientific Software, Chicago, IL); a non-linear one-site binding model was used to calculate Bmax and K_D_ values^10^. Data show means (n ≥ 3); SEM are shown (Supplemental Fig. 2).

### Data processing

The raw fluorescence data for the FLIPR assays were interpreted as a ratio of Max/Min values which were normalized to positive and negative controls to give percent response scores. The Max refers to the maximum response over the kinetic read in the FLIPR (140 s). The Min refers to the minimum or basal response in the FLIPR prior to the addition of GnRH. The raw fluorescence data for the TR-FRET assay was interpreted as a ratio of the fluorescence response at 618 nM and 671 nM when excited at 340 nM. Dose–response curves were fit using a four-parameter variable slope sigmoidal. The reported EC_50_ values were generated from fitted curves by solving for the X-intercept value at the 50% activity level of the Y-intercept value. The following rule was used to declare a compound as “active” or “inactive”: compounds with EC_50_ > 5 µM were considered inactive. Compounds with EC_50_ ≤ 5 µM were considered active.

### Statistics and graphics for IP

All HTS data was qualified using Z-factor (Z′) analysis which is a unitless statistic that is used to determine assay robustness. When Z′ is greater than 0.5 the assay is considered excellent^11^. Data (> 3) were analyzed with one-way analysis of variance and then paired Student’s t-test (SigmaStat 3.1; Jandel Scientific Software); *P < 0.05 was considered significant. SigmaPlot 12.5 (Jandel Scientific) was used to analyze the IP and binding data.

### Screening library

The Scripps Diversity Drug Library (SDDL), used for this HTS campaign, currently consists of 646,275 unique and individually pure compounds, representing a wide diversity of drug-like small organic molecules used in traditional and non-traditional drug-discovery biology. The SDDL has been curated from over 20 commercial sources, supplemented with academic sources, including compounds and sub-libraries prepared internally, and thus ~ 40,000 compounds in the collection are unique to Scripps. SDDL compounds were selected based on scaffold novelty, physical properties (i.e., “drug-likeness”) and spatial connectivity. In its current state, the SDDL has several focused sub-libraries for screening popular drug-discovery target classes (e.g., kinases/transferases, GPCRs, ion channels, nuclear receptors, hydrolases, transporters), with diverse chemistries (e.g., click-chemistry, PAINS-free collections, Fsp^3^ enriched, covalent inhibitors and natural product collections) and with desirable physical properties (“rule-of-five”, “rule-of-three”, polar surface area, etc.)^12–17^. All samples in the SDDL were confirmed for purity via LC–MS and/or NMR to provide adequate QA/QC after completion of an HTS campaign.

### 10 K pilot for control compound identification

**Figure 3:**
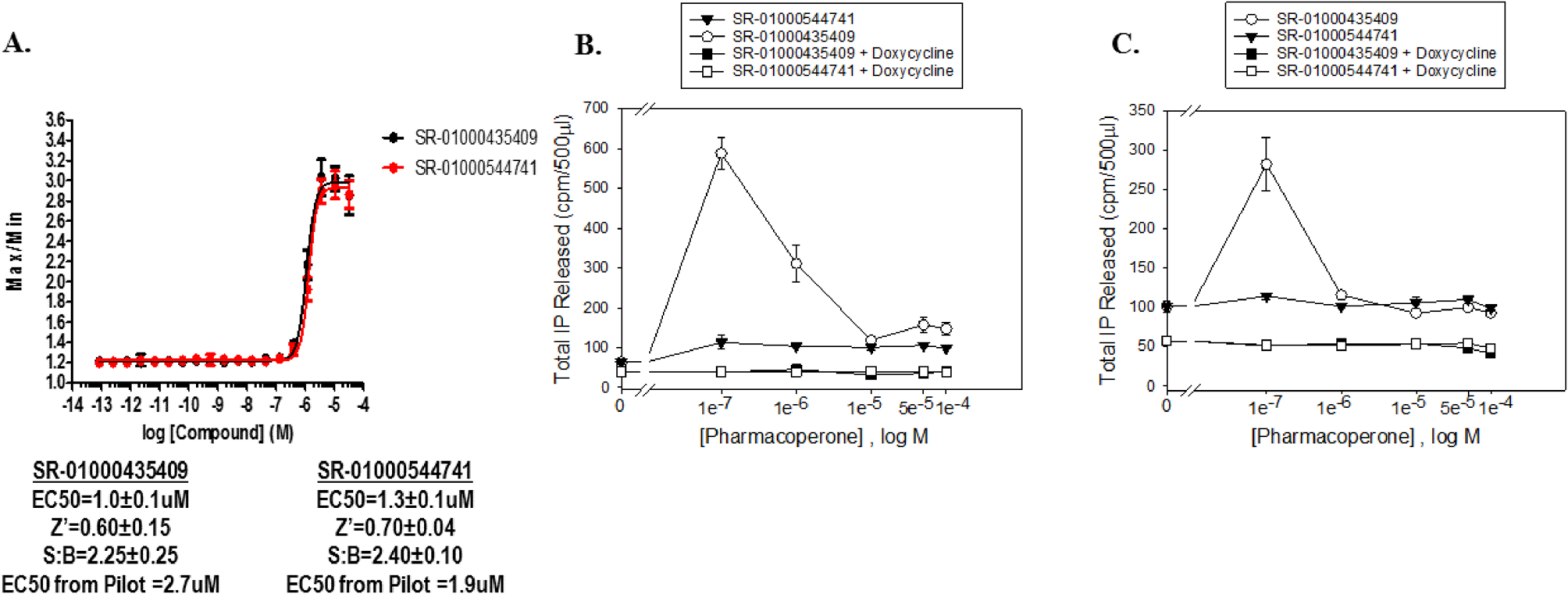
Positive control validation. Validation of 2 active compounds for use as HTS positive controls. (**A**) Dose–response curves of the compounds in the Fluo2 HTS format. Statistics for the compounds are found below the graph. (**B**) IP assay for the activity of the GnRHR E90K on the various control compounds in the presence or absence of doxycycline. (**C**) IP assay for the activity of the WT GnRHR on the various control compounds in the presence or absence of doxycycline.

Initially, a 10,000-compound (10 K) pilot screen was implemented for two reasons. First, we needed to make sure we could identify compounds that could restore the GnRHR E90K mutant function since we originally found no hits in a formerly executed and published LOPAC screen^7^. Second, IN3 was not available to use in this HTS campaign, so we needed to find other compounds that may be useful as positive controls. The 10 K pilot screen test compounds were pulled from the SDDL. The results of the pilot screen can be found in Supplemental Fig. 1. From the pilot screen, 12,801 compounds were tested in triplicate at 6 µM nominal concentration of which 89 compounds confirmed activity. From these we identified 11 compounds that had EC_50_ < 10 µM when tested in 10-point 1:3 dilution series. Two compounds were further characterized for their responses in the HTS assay as well as a secondary IP assay, and are shown in Fig. 3. SR-01000435409 showed activity in all three assay formats and was utilized as the positive control for the HTS assays going forward. We also included SR-01000544741 as a reference control on each plate as well.

### Cytotoxicity assay

This assay was adopted from our work done as part of a former publication albeit using an alternative cell line^18^. HEK cells were cultured in T-175 cm^2^ flasks at 37 °C and 95% relative humidity (RH). The growth media consisted of Dulbecco's Modified Eagle's Media (DMEM) containing 10% v/v fetal bovine serum and 1X antibiotic mix (penicillin, streptomycin, and neomycin).

Prior to the start of the assay, 500 cells in a 5 μL volume of growth media were dispensed into each well of 1536-well tissue culture-treated microtiter plates. The assay was started immediately by pintool transfer of 28 nL of test compound in DMSO (0.06% final DMSO concentration), DMSO alone, or doxorubicin (150 µM final concentration) to the appropriate wells. Next, the plates were incubated for 48 h at 37 °C (5% CO_2_, 95% RH). After equilibrating the plates to room temperature for 30 min, the assay was stopped by dispensing 5 μL of CellTiter-Glo reagent to each well, followed by incubation at room temperature for 15 min. Well luminescence was then measured on the ViewLux plate reader.

The percent inhibition for each compound was calculated using the following controls: Test_Compound is defined as wells containing test compound. Low_Control is defined as wells containing DMSO. High_Control is defined as wells containing doxorubicin.

## Results

### Primary HTS

**Figure 4:**
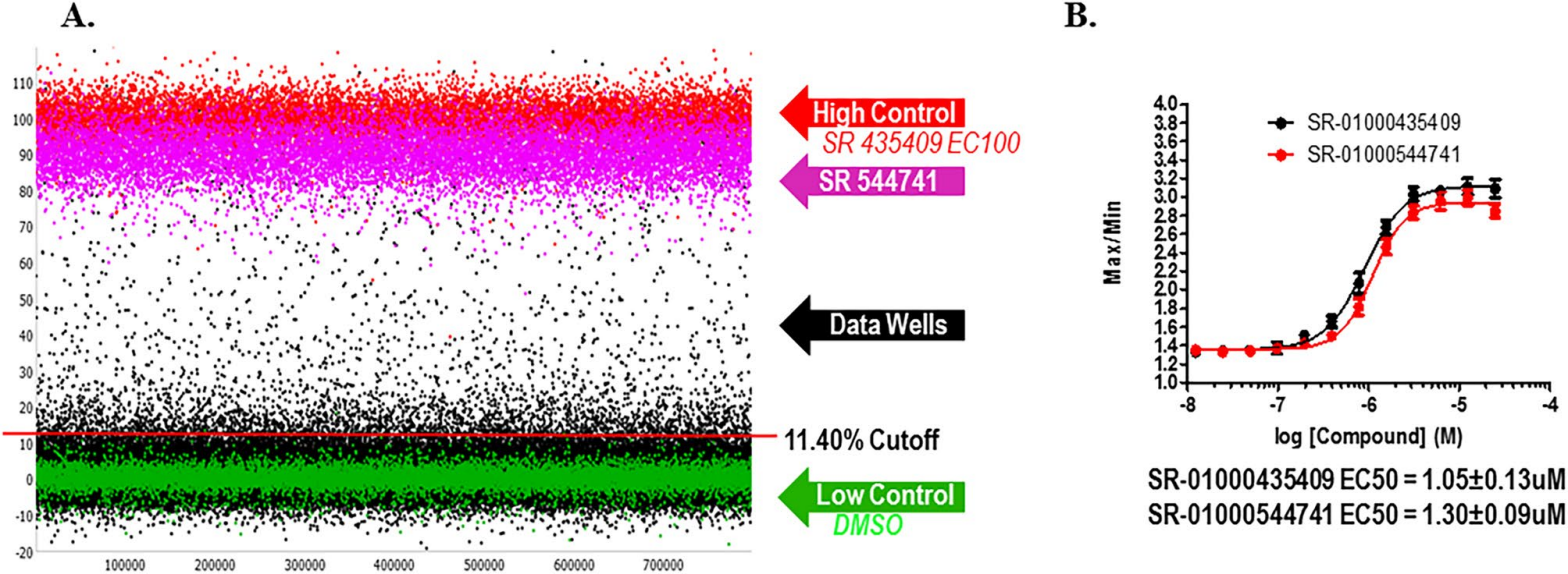
GnRHR primary HTS. Primary HTS campaign scatterplot and control compound dose responses. (**A**) The scatterplot of all compounds tested along with all controls. (**B**) The dose response curves for the reference compounds tested as controls for the assay. N = 16 replicates per concentration; error bars presented in SD.

In the primary screen, the remainder of the 646,275 SDDL compounds were tested in singlicate using the optimized conditions described earlier. The results of the primary screen are shown in Fig. 4 and the statistics can be found in Table 1. In summary, 4,820 compounds were found to be active, i.e., they exhibited activity greater than a cutoff of 11.40%, a value obtained using an interval based mathematical algorithm^19,20^. Day to day reproducibility was monitored using the SR-01000435409 and SR-01000544741 concentration–response curves to ensure their EC_50_ for rescue was within threefold of their average. The overall Z′ for the primary screen was 0.8 ± 0.1 with a S:B of 2.7 ± 0.3. The pEC_50_ of the SR-01000435409 was stable at − 5.97 ± − 7.88, and the EC_50_ of the SR-01000544741 was also stable at − 5.88 ± − 7.04 over 20 plates throughout the primary screen (Fig. 4B).

**Table 1:** GnRHR HTS campaign.

Step	Screen type	Target	Number of compounds tested (5.2 μM)	Selection criteria	Number of selected compounds (hit rate)	Assay statistics	
						Z′	S/B
1a	Primary screen	GNRHR	646,275	11.40%^a^	4,820 (0.75%)	0.79 ± 0.06	2.46 ± 0.26
2a	Confirmation screen	GNRHR	4,809	11.40%^b^	1,668 (34.7%)	0.76 ± 0.03	2.42 ± 0.04
2b	Confirmation screen (IPOne)	GNRHR	4,809	21.87%^c^	812 (16.9%)	0.78 ± 0.02	1.72 ± 0.01
2c	Counterscreen screen	GNRHR + DOX	4,809	41.6 %^d^	32 (0.67%)	0.81 ± 0.02	2.83 ± 0.05
3a	Dose response screen	GNRHR	662	EC50 < 5µM	420 (63%)	0.75 ± 0.03	2.32 ± 0.06
3b	Dose response screen (IPOne)	GNRHR	662	EC50 < 5µM	466 (70%)	0.79 ± 0.03	1.60 ± 0.02
3c	Dose response counterscreen	GNRHR + DOX	662	EC50 < 5µM	0 (0%)	0.77 ± 0.06	2.31 ± 0.21
4a	Powder confirmation (Fluo2)	GNRHR	74	EC50 < 5µM	70 (95%)	0.77 ± 0.034	1.65 ± 0.026
4b	Powder confirmation (Fluo2)	GNRHR + DOX	74	EC50 < 5µM	0 (0%)	0.66 ± 0.06	2.11 ± 0.02

### Confirmation and counterscreen assays

After completion of the primary HTS, all compounds were selected for cherry-picking, of which 4,809 were available for testing in the secondary assays. The confirmation screen used the same reagents and detection system as the primary screening assay, but tested each of the compounds at a single concentration (nominally 5.2 µM) in triplicate. The GnRHR FLUO2 pharmacoperone confirmation assay performance was excellent with an average Z′ of 0.8 ± 0.03 and a S:B of 2.4 ± 0.04. Using the primary assay cut-off of 11.4% response, 1,668 hits confirmed activity equal to 35%.

The GnRHR IP-One TR-FRET orthogonal confirmation assay performance was also excellent with an average Z′ of 0.8 ± 0.02 and a S:B of 1.7 ± 0.01. Using a standard HTS assay cut-off of 21.9% response, which was derived from the activity of all DMSO wells tested + 3SD, 812 hits confirmed activity equal to 17%.

**Figure 5:**
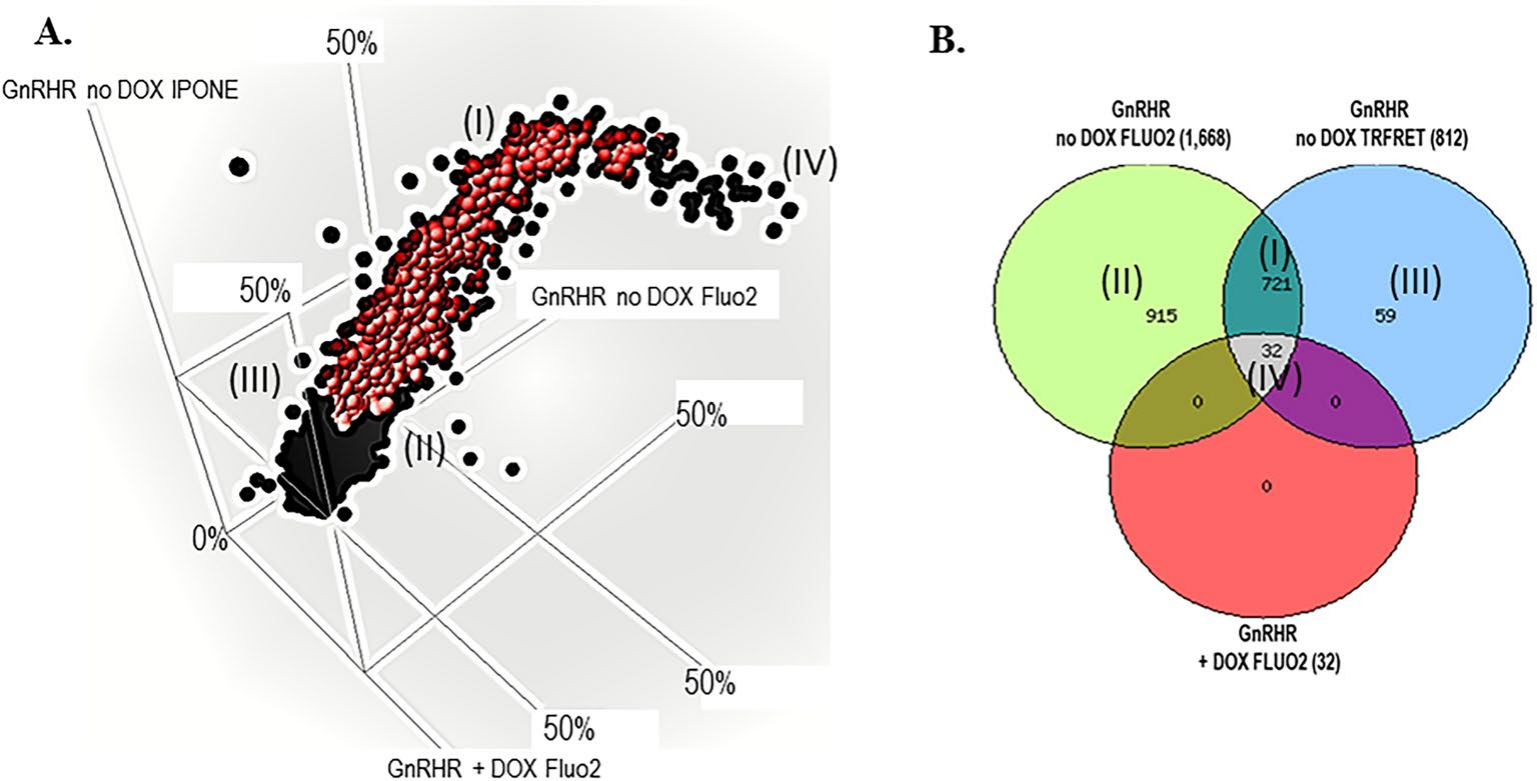
GnRHR confirmation and counterscreen. (**A**) The 3-way correlation plot of all compounds tested in all three assay formats represented as average percent response. The red circles represent the 721 selective compounds that are shown in the indicated section of the Venn diagram (I). (**B**) A Venn diagram representing the active compounds of the three assays. The 721 represents compounds that are active in the GnRHR no DOX Fluo2 assay and GnRHR no DOX IP-One assay and inactive in the GnRHR “+” DOX Fluo2 counterscreen. Each section of active compounds in the Venn diagram are represented by roman numerals, and the matching area of the 3D correlation plot are indicated.

The “+” DOX counterscreen assay performance was also excellent with an average Z′ of 0.8 ± 0.02 and a S:B of 2.8 ± 0.05. In this assay, cells are pretreated with doxycycline for 36 h to block receptor expression. However, the control SR-01000435409 elicits a minor response in the “+” DOX treated cells which is about 40% of that observed in the DOX “−“ cells. This is presumably due to off target receptor activation which is triggering a partial Ca^2+^ response. In this case we utilized this opportunity to define a reasonable hit cut-off to help preserve compounds to move forward for titration testing. We employed a hit cut-off derived from average response of the “+” DOX treated cells in the presence of SR-01000435409 EC100 (10 µM) + 3SD as the cut-off which equates to 41.6%. A total of 32 hits were found (0.67%). These 32 hits presumably hit an off target within the screening system and weren’t considered of further interest. A 3D scatterplot from each confirmation and counterscreen assays and the representative Venn diagram are found in Fig. 5. Using well established and previously utilized in-silico tools such as PAINs and promiscuity filtering, we identified 666 compounds active in < 5 other screens that were also free from PAINs^17,21–23^. Of these, 662 of these compounds were available for cherry-picking (Table 1).

### Titration and counterscreen assays

The titration assays were run following the confirmation and counterscreening assay protocols with 662 compounds tested at ten doses (threefold dilutions) in triplicate. The GnRHR FLUO2 titration assay performance was excellent with an average Z′ of 0.75 ± 0.03 and a S:B of 2.32 ± 0.06. The GnRHR IP-One TRFRET titration assay performance was excellent with an average Z′ of 0.79 ± 0.03 and a S:B of 1.60 ± 0.02. The GnRHR FLUO2 “+” DOX counterscreen titration assay performance was excellent with an average Z′ of 0.77 ± 0.06 and a S:B of 2.31 ± 0.21. For each tested compound, percent activation was plotted against compound concentration. Of those tested, 399 compounds demonstrated an EC_50_ < 5 µM in the GnRHR no DOX FLUO2 pharmacoperone assay and in the GnRHR no DOX IP-One TRFRET pharmacoperone assay with an EC_50_ > 5 µM in the GnRHR FLUO2 “+” DOX counterscreen. All titration assay results are tabulated in Table 1. Structural analysis along with correlation of the EC_50_ data from both no DOX assays was done to help select a panel of molecules for secondary mode of action studies (MOA) and a table of the structural classes of the compounds was selected for further mechanism of action studies is shown in Table 2.

**Table 2:** GNRHR molecules of interest for MOA studies.

Compound #	Bmax fmol (per 10^5^cells)	Kd (nM)	Receptor Density on the plasma membrane (10^6^ molecules per cell)	IP Rescue EC50 (μM)	Cytotoxicity EC50 (μM)	Structure	Structure class
SR-01000434453-2	29 ± 0.6	9 ± 0.3	0.22 ± 0.06	1.04	> 59.6		Cyanoacrylates
SR-01000273592-2	29 ± 1	3 ± 0.3	0.17 ± 0.04	2.08	> 59.6		Cyanoacrylates
SR-05000713815-1	10 ± 0.5	3 ± 0.4	0.07 ± 0.02	8.22	> 59.6		2-Aminobenzoyl benzimidazoles/benzothiazoles
SR-01000567813-2	21 ± 0.9	8 ± 0.7	0.13 ± 0.03	0.34	20.7		Other series (single hit)
SR-01000012920-2	5 ± 0.6	2 ± 0.7	0.03 ± 0.009	2.05	32.6		2-Aryl imidazo pyridines and related compounds
SR-01000521119-2	5 ± 0.7	1 ± 0.6	0.03 ± 0.01	1.79	> 59.6		Other series (single hit)
SR-01000211348-2	8 ± 3	12 ± 8	∞	4.38	28.8		Benzo indolones
SR-01000255670-3	14 ± 3	7 ± 3	0.099 ± 0.05	2.92	> 59.6		Spaced diamines
SR-05000713804-1	0.09 ± 0.2	6.2733e − 10 + 2	∞	4.75	> 59.6		2-Aminobenzoyl benzimidazoles/benzothiazoles
SR-01000434452-2	∞	∞	∞	8.73	> 59.6		Cyanoacrylates
SR-01000439482-2	11 ± 0.2	6 ± 0.2	0.07 ± 0.01	0.98	15.9		2-Aryl imidazo pyridines and related compounds
SR-01000435409	14 ± 3	12 ± 5	0.09 ± 0.01	4.93	ND		Other series (control Compound)

All compounds tested for concentration response were submitted for LC–MS analysis. 648 samples (97.9%) demonstrated the expected molecular mass. As determined by nominal methods (UV–Vis spectroscopy, MS and ELSD), 591 samples demonstrated purity of > 80%.

### Chemical optimization and MOA

Following HTS, medicinal chemistry was done iteratively to first confirm activity using independently-acquired or internally-synthesized samples and then to determine if the molecules were active as pharmacoperones that rescue receptor activity but behave as a neither an agonist nor an antagonist at the cognate ligand-binding site. Powders for 74 compounds representative of the structural classes shown in Table 2 were either purchased or synthesized for testing in the original primary and counterscreen assays. All molecules reconfirmed potency in each of the assays. Based on the criteria of achieving an EC_50_ < 5 µM, 70 out of 74 molecules were considered active in the primary and IP-One assays and were inactive in the “+” Dox counterscreen. This prompted an additional round of medicinal chemistry including testing 91 additional molecules that were either purchased or synthesized as powders. Based on the same criteria, six molecules of interest appeared to be active and selective. The disparity in initial activity confirmation (70 out 74) that seemed to diminish for the second round of molecules (6 out of 91) led to some concern. To address this, the top 43 molecules from the first and second round were retested in the three assays and the results were deemed consistent from prior results, with all EC_50_s at < threefold deviation from before. To confirm integrity of the samples, LC–MS was performed for each of the medicinal chemistry derived analogs and found to be at the expected mass and > 80% purity.

**Figure 6:**
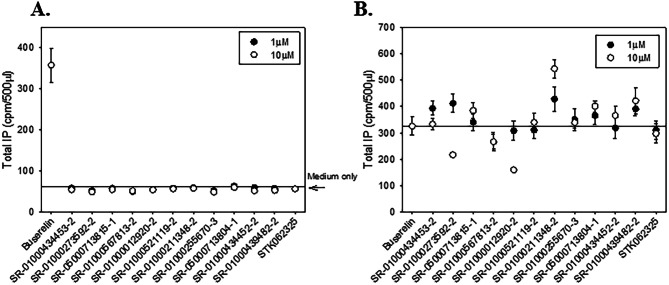
Wildtype GnRHR agonist and antagonist IP assays and mutant rescue. (**A**) IP assay of agonist activity of 12 compounds on the WT GnRHR. (**B**) IP assay of antagonism of the 12 compounds on the WT GnRHR. All experiments were done at least three times and averaged; N of 3 ± SEMs.

From these data the most potent and efficacious molecules from each class were selected for low throughput total inositol phosphate and radioligand binding assays as shown in Table 2. These assays were run testing 11 molecules in total with STK062325 as a control. In this assay, wildtype GnRHR stably expressed in HeLa cells were tested as described above. The purpose of this assay is to expose molecules that may act as agonists or antagonists when tested in the absence or presence of Buserelin, the cognate receptor ligand, respectively. The goal is to identify molecules that are devoid of any agonist nor antagonist activity in this sense because ultimately using a compound that both rescues mutant receptor functionality and acts by eliciting a response at the cognate receptor (agonist activity) or has strong antagonist activity and must be “washed out” limits the therapeutic reach; a fate that ultimately demised the clinical utility of IN3^24^. All 11 compounds were tested at both 1 and 10 µM in the IP assay for agonist or antagonist activity. None of the test compounds exhibited appreciable agonist nor antagonist activity (Fig. 6A,B) while the formerly identified controls SR-01000435409 did elicit an antagonist response (not shown). Rescue potential for the GnRHR E90K mutant was checked for all 11 compounds using the stably transfected HeLa cells. All showed rescue (Supplemental Fig. 3). Ten-point concentration curves of 1:3 dilutions were determined in the IP assay to calculate the EC_50_ for each compound; the EC_50_s ranged from 0.345 µM (lowest) up to the highest of 8.7 µM (Table 2).

Supplemental Fig. 2 shows saturation and Scatchard binding activity of the 11 compounds as well as the control. Bmax, Kd and average numbers of receptors at the membrane were determined. The SR-01000434453-2 compound showed very high Bmax, Kd and receptor molecules and did not show saturation with the radioactive ligand, concluding that this would not be a therapeutic choice for use of the compound even though there is rescue of E90K. The SR-05000713815-1 compound did not saturate with ^125^I-Buserelin, which would make for a questionable therapeutic use of this compound. The other compounds show promise for use in a therapeutic effort with Bmax and Kd values comparable to the previously published IN3^25^. The Bmax and Kd values from the 11 compounds, along with the control compound can be found in Table 2.

Note that activity in any of the aforementioned assays could be also due to general cytotoxicity of the molecules to the cells. Hence, we tested the same 11 molecules in concentration–response profiles for their cytotoxic effect on HEK 293 cells. None appeared significantly toxic with the most potent CC_50_ being 16 µM and all others with a CC_50_ > 30 µM. Doxorubicin was used as a control with an EC_50_ of 0.1 µM. Compounds were tested in a 10-point dose–response in triplicate starting at 59.6 µM. Z′ = 0.76 ± 0.05, S:B = 3.31 ± 0.09 over three plates.

This provided evidence for the first time that we had identified specific and reproducible pharmacoperones that rescue GnRHR 90 K mutant activity without interacting with the cognate receptor ligand binding site.

## Discussion

In many of our previous studies, we have demonstrated the efficacy of GnRHR-specific pharmacoperones for rescuing misfolded GnRHR mutants from ER retention, leading to plasma membrane localization and normal signaling activity using cell culture. GnRHR-specific pharmacoperones come from several different chemical classes^4^ which presents the opportunity for a broad therapeutic reach in the treatment of diseases caused by misfolded proteins. The issue with these former molecules is that they were all antagonists of the receptor, thereby limiting their therapeutic potential.

Our HTS campaign identified eight different structural classes that showed rescue of the GnRHR E90K mutant. Because these are not agonists nor antagonists, the binding of these pharmacoperones will not interfere with the binding site of the native GnRH ligand making them more desirable for use in-vivo and a washout of the pharmacoperone will not be as critical.

At the completion of the titration assays, 399 compounds were identified that selectively rescue the effect of GnRHR on the release of intracellular calcium and increase of IP3 in cells containing the mutated GnRHR gene at EC_50_ < 5 µM. To determine which compounds to progress with for powder testing, we first focused on the 174 most potent compounds. These compounds had EC_50_ < 2 µM in the GnRHR no DOX Fluo2 titration assay. Next unstable or reactive compounds with high MW (> 500) and a high logP (> 5) were eliminated. We also eliminated the compounds that were not commercially available. Finally, we removed compounds with low efficacy (50–55%) and low potency (1.5–2 µM). This left 74 compounds to be tested as powders. Fresh powdered samples of these compounds were obtained and tested in the titration assays. 95% (70 compounds) confirmed activity (Table 1) using the same procedure tested during the HTS campaign.

The 74 compounds were further broken down in structural classes to determine which compounds would move forward to secondary assays. Twenty compounds were selected to test for agonist and antagonist activity in stably transfected HeLa cells with wildtype (WT) GnRHR. The 11 compounds were chosen from the highest activity compounds from eight structural classes (Table 2). These compounds were tested in the WT GnRHR agonist and antagonist IP assay. None of the 11 compounds tested showed appreciable agonist or antagonist effect (Fig. 6).

This is significant because we have successfully completed an HTS campaign, identifying potential pharmacoperones capable of rescuing GnRHR activity in cells expressing the E90K GnRHR misfolded protein. We have shown that, in this system, we can restore the function of the GnRHR by demonstrating GnRH stimulation of calcium release and IP3 accumulation in these mutant cell lines in the presence of a pharmacoperone. Most of these potential pharmacoperones showed activity with fresh, independent samples, which were also inactive in the counterscreen. They fall into one of eight structure classes and appear to set the path for chemically tractable lead synthesis, as all were found to be non-promiscuous and PAINS free. Initial efforts have determined that these compounds do not act as agonists or antagonists in cells expressing the wildtype GnRHR protein. Those that show no antagonist characteristics could be interesting leads for therapeutic intervention. We plan to test these leads for in-vitro and subsequently in-vivo DMPK testing. Based upon those results we will pursue further chemistry initiatives to optimize lead molecules prior to utilization in animal models of the associated disease. This HTS campaign demonstrates the effective use of appropriate assays and protocols to identify potential pharmacoperones which ultimately may be useful in the treatment of diseases caused by protein misfolding and misrouting, including hypogonadotropic hypogonadism or Kallmann’s syndrome.

## Supplementary information

Supplementary file1 (DOCX 505 kb)

## Data Availability

We will make materials, data and associated protocols promptly available to readers upon request.

## References

[1] Conn PM, Janovick JA (2009). Drug development and the cellular quality control system. Trends Pharmacol. Sci..

[2] Janovick JA, Maya-Nunez G, Conn PM (2002). Rescue of hypogonadotropic hypogonadism-causing and manufactured GnRH receptor mutants by a specific protein-folding template: Misrouted proteins as a novel disease etiology and therapeutic target. J. Clin. Endocrinol. Metab..

[3] Conn PM, Ulloa-Aguirre A (2010). Trafficking of G-protein-coupled receptors to the plasma membrane: Insights for pharmacoperone drugs. Trends Endocrinol. Metab..

[4] Perrett RM, McArdle CA (2013). Molecular mechanisms of gonadotropin-releasing hormone signaling: Integrating cyclic nucleotides into the network. Front. Endocrinol. (Lausanne).

[5] Janovick JA (2003). Structure-activity relations of successful pharmacologic chaperones for rescue of naturally occurring and manufactured mutants of the gonadotropin-releasing hormone receptor. J. Pharmacol. Exp. Ther..

[6] Ulloa-Aguirre A, Zarinan T, Dias JA, Conn PM (2014). Mutations in G protein-coupled receptors that impact receptor trafficking and reproductive function. Mol. Cell. Endocrinol..

[7] Conn PM (2014). A phenotypic high throughput screening assay for the identification of pharmacoperones for the gonadotropin releasing hormone receptor. Assay Drug Dev. Technol..

[8] Brothers SP (2002). Conserved mammalian gonadotropin-releasing hormone receptor carboxyl terminal amino acids regulate ligand binding, effector coupling and internalization. Mol. Cell. Endocrinol..

[9] Brothers SP, Janovick JA, Conn PM (2003). Unexpected effects of epitope and chimeric tags on gonadotropin-releasing hormone receptors: Implications for understanding the molecular etiology of hypogonadotropic hypogonadism. J. Clin. Endocrinol. Metab..

[10] Klotz IM (1982). Numbers of receptor sites from Scatchard graphs: Facts and fantasies. Science.

[11] Zhang JH, Chung TD, Oldenburg KR (1999). A simple statistical parameter for use in evaluation and validation of high throughput screening assays. J. Biomol. Screen.

[12] Lovering F, Bikker J, Humblet C (2009). Escape from flatland: Increasing saturation as an approach to improving clinical success. J. Med. Chem..

[13] Lipinski CA, Lombardo F, Dominy BW, Feeney PJ (2001). Experimental and computational approaches to estimate solubility and permeability in drug discovery and development settings. Adv. Drug Deliv. Rev..

[14] Kolb HC, Sharpless KB (2003). The growing impact of click chemistry on drug discovery. Drug Discov. Today.

[15] Ding S, Gray NS, Wu X, Ding Q, Schultz PG (2002). A combinatorial scaffold approach toward kinase-directed heterocycle libraries. J. Am. Chem. Soc..

[16] Congreve M, Carr R, Murray C, Jhoti H (2003). A 'rule of three' for fragment-based lead discovery?. Drug Discov. Today.

[17] Baell JB, Holloway GA (2010). New substructure filters for removal of pan assay interference compounds (PAINS) from screening libraries and for their exclusion in bioassays. J. Med. Chem..

[18] Spicer TP (2019). Identification of antimalarial inhibitors using late-stage gametocytes in a phenotypic live/dead assay. SLAS Discov..

[19] Jambrina E (2016). An integrated approach for screening and identification of positive allosteric modulators of *N*-methyl-d-aspartate receptors. J. Biomol. Screen.

[20] Smith E (2016). Identification of potential pharmacoperones capable of rescuing the functionality of misfolded vasopressin 2 receptor involved in nephrogenic diabetes insipidus. J. Biomol. Screen.

[21] Spicer T (2014). Identification of potent and selective inhibitors of the *Plasmodium falciparum* M18 aspartyl aminopeptidase (PfM18AAP) of human malaria via high-throughput screening. J. Biomol. Screen.

[22] Smith E (2015). Application of parallel multiparametric cell-based FLIPR detection assays for the identification of modulators of the muscarinic acetylcholine receptor 4 (M4). J. Biomol. Screen.

[23] Smith E (2017). A homogeneous cell-based halide-sensitive yellow fluorescence protein assay to identify modulators of the cystic fibrosis transmembrane conductance regulator ion channel. Assay Drug Dev. Technol..

[24] Conn PM, Spicer TP, Scampavia L, Janovick JA (2015). Assay strategies for identification of therapeutic leads that target protein trafficking. Trends Pharmacol. Sci..

[25] Janovick JA, Pogozheva ID, Mosberg HI, Cornea A, Conn PM (2012). Rescue of misrouted GnRHR mutants reveals its constitutive activity. Mol Endocrinol.

