# Supplementary material for: Rescue of mutant gonadotropin-releasing hormone receptor function independent of cognate receptor activity
Source: Sci Rep. 2020 Jun 29;10:10579. doi: 10.1038/s41598-020-67473-w (PMC7324376; doi:10.1038/s41598-020-67473-w)
Supplement: Supplementary file 1 — Supplementary file1 (DOCX 505 kb) [file 41598_2020_67473_MOESM1_ESM.docx]

**Rescue of Mutant Gonadotropin-Releasing Hormone Receptor Function Independent of Cognate Receptor Activity**

**Emery Smith^1a†^, Jo Ann Janovick^2†^, Thomas D. Bannister^1b^, Justin Shumate^1a^, Vadivel Ganapathy^2^, Louis Scampavia^1a^ and Timothy P. Spicer^1a^***

Affiliations:

^1^The Scripps Research Molecular Screening Center, Department of Molecular Medicine^1a^ and Department of Chemistry^1b^, Scripps Research Florida, 130 Scripps Way, Jupiter, Florida, USA

^2^Texas Tech University Health Sciences Center, 3601 4th Street, Lubbock, Texas, USA

**Short title:** Gonadotropin-Releasing Hormone Receptor Pharmacoperones

Key words: calcium signaling, pharmacoperone, GPCR, protein folding, protein trafficking

**^†^**Equal Contribution

**Correspondence:* [spicert@scripps.edu](mailto:spicert@scripps.edu)

*Timothy P. Spicer
Scripps Research
130 Scripps Way #1A1
Jupiter, FL 33458
U.S.A.*

*561-228-2150*

**
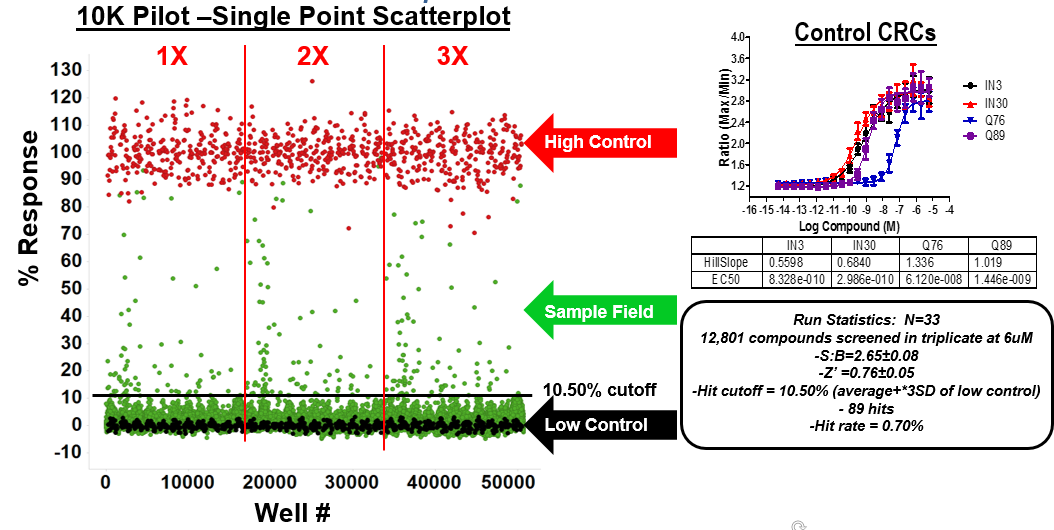
**

**Supplemental Figure 1. GnRHR 10K pilot.** 10K pilot screen run in triplicate at a single concentration. The scatterplot of the 12,801 compounds run in triplicate at 6 µM screening concentration. Z’ for the pilot was 0.76±0.05, S:B was 2.65±0.08, N=33 plates. 89 active compounds with an average activity greater than 10.5% (average+3SD of the low control).

**Supplemental Figure 2A.**

**Supplemental Figure 2B.**

**Supplemental Figure 2. Saturation binding and Scatchard plots of the 11 compounds.** E90K GnRHR mutant binding after rescue with each of the 11 compounds. All experiments were done at least 3 times and averaged; N of 3 + SEMs. **A.** Saturation Binding Plots. **B.** Scatchard Plots.


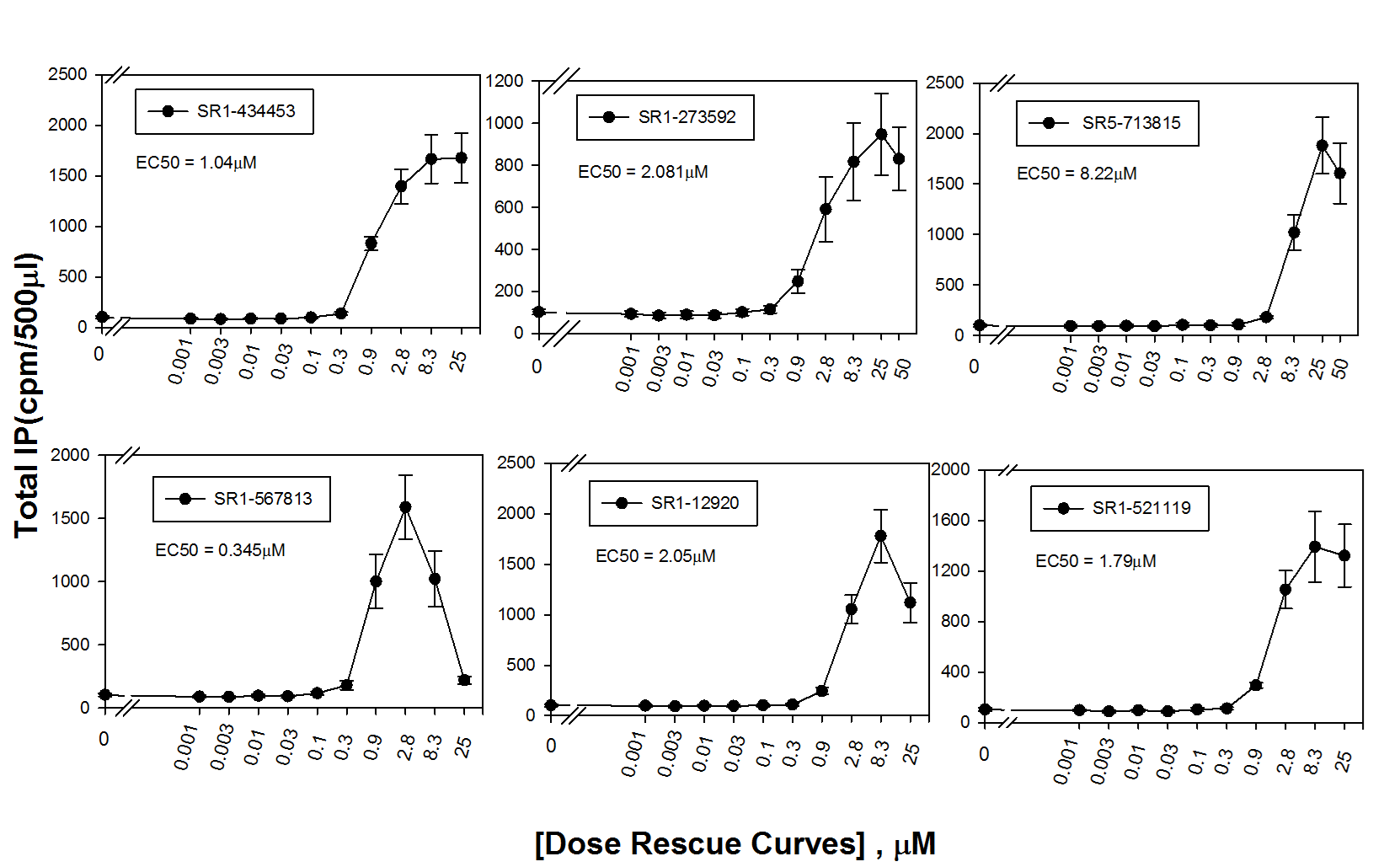

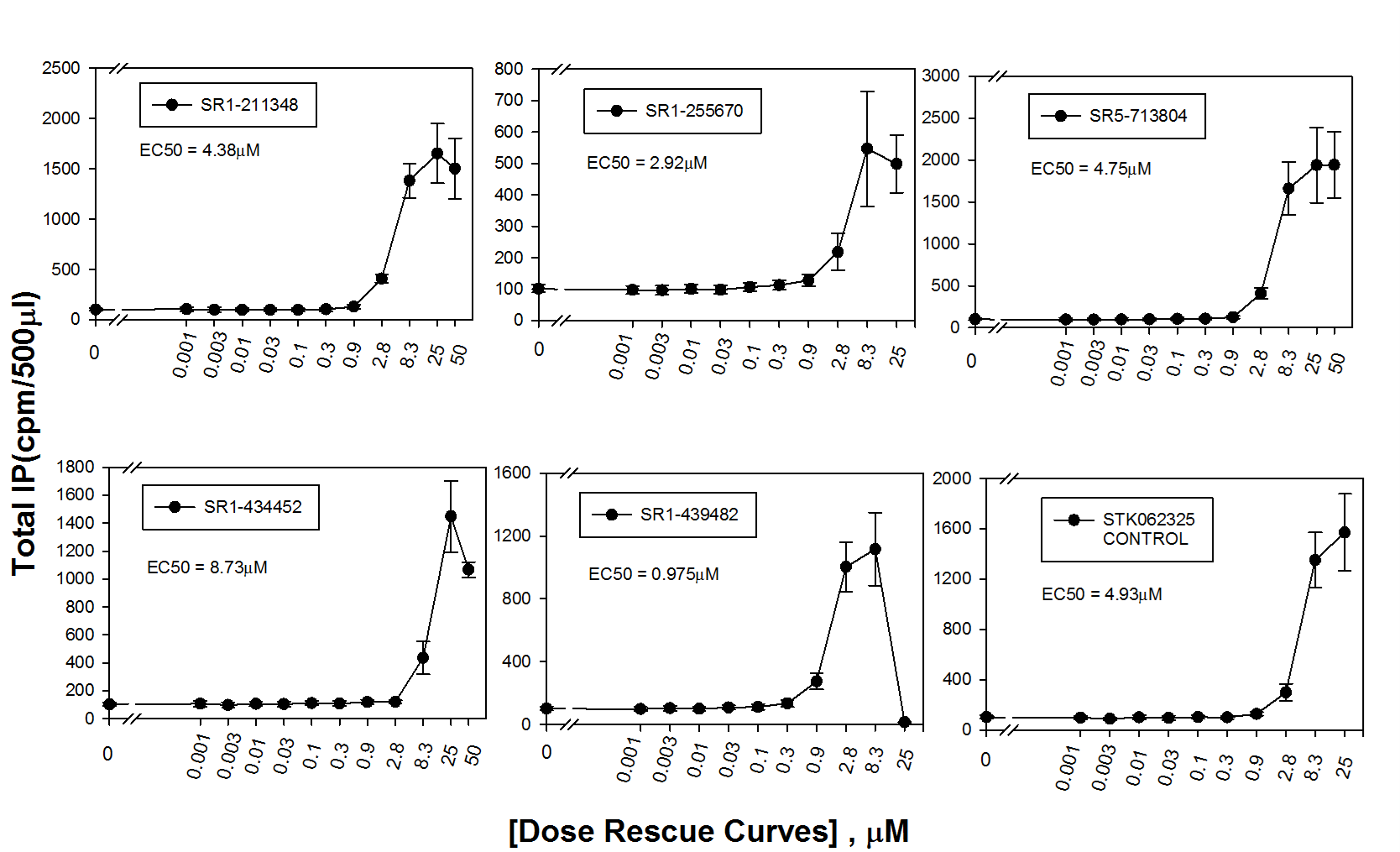


**Supplemental Figure 3: GnRHR IP assay mutant rescue.** IP assay activity of 12 compounds showing rescue of the E90K GnRHR mutant. All experiments were done at least 3 times and averaged; N of 3 + SEMs.


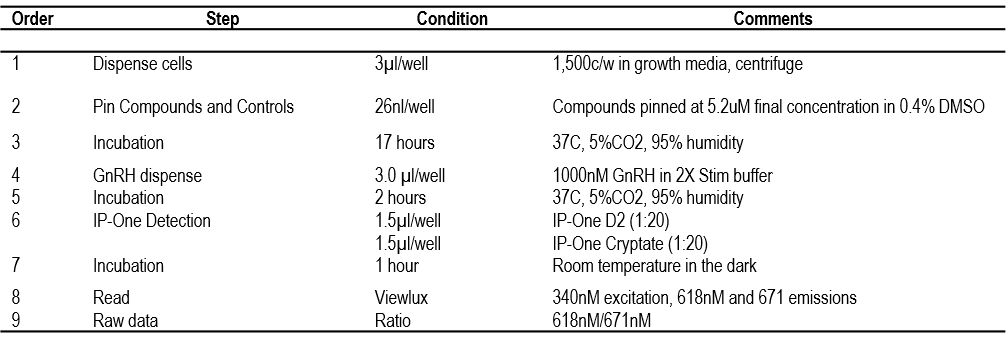

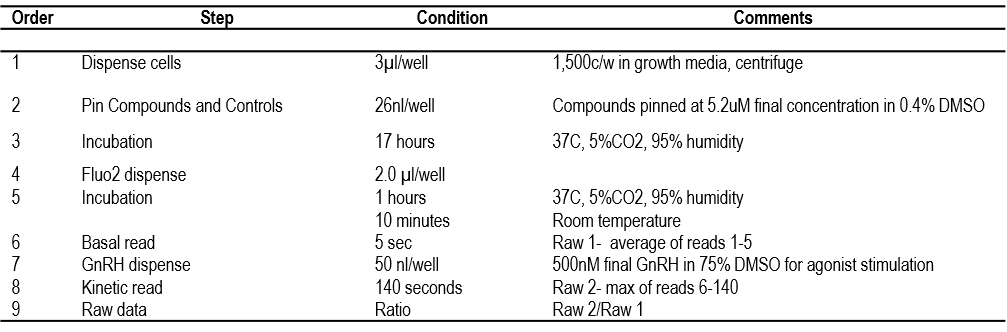


***Supplemental Table 2. GnRHR IP-One TR-FRET assay protocol in 1,536-well plate format.***

***Supplemental Table 1. GnRHR FLUO2 assay protocol in 1,536-well plate format.***
